# Precursor Lesions, Overdiagnosis, and Oral Cancer: A Critical Review

**DOI:** 10.3390/cancers16081550

**Published:** 2024-04-18

**Authors:** Nicola Cirillo

**Affiliations:** 1 Melbourne Dental School, Faculty of Medicine, Dentistry and Health Sciences, The University of Melbourne, Carlton, VIC 3053, Australia; nicola.cirillo@unimelb.edu.au; 2 School of Dentistry, University of Jordan, Amman 11733, Jordan; 3 CoTreatAI, CoTreat Pty Ltd., Melbourne, VIC 3000, Australia

**Keywords:** lip and oral cavity cancer, cancer mortality, OPMDs, overdiagnosis, education

## Abstract

**Simple Summary:**

This article discusses a common approach to the early detection of oral cancer, which focuses on identifying and monitoring certain mouth conditions, known as oral potentially malignant disorders (OPMDs). However, despite this strategy, the death rates from cancers of the lip and mouth have not improved in 30 years. Surprisingly, only around 10% of oral cancers actually start as premalignant, and most OPMDs rarely turn into cancer. The article suggests that only a few specific types of these disorders, which have a higher risk of becoming cancerous and/or are prevalent in populations, really need intervention. It also questions the effectiveness of regarding OPMDs as heralding malignancy and calls for a different approach to reduce deaths for oral cancer.

**Abstract:**

Despite the profession placing great emphasis on oral potentially malignant disorders (OPMDs) as a gateway for early recognition and consequently better outcomes for oral cancer, the death rates for lip and oral cavity cancer have remained stagnant for three decades. Evidence shows that only a small fraction of oral cancers are in fact preceded by OPMDs, and that most OPMDs have an annual transformation rate of less than 1%. As OPMDs encompass a very heterogeneous group of oral conditions, it could be argued that only patients with oral mucosal diseases bearing a substantial risk of malignant transformation warrant close surveillance and treatment, these include proliferative leukoplakia, erythroplakia, non-homogeneous leukoplakia, as well as diseases presenting with severe dysplasia at biopsy. In this narrative review, I discuss the intricate epidemiology of the malignancies that we colloquially refer to as oral cancer, explore the limitations of focusing on OPMDs to reduce the incidence and mortality of oral cavity cancer, and argue that a *may-be* cancer label represents overdiagnosis for most OPMDs.

## 1. Introduction

The goal of early cancer diagnosis is to reduce cancer morbidity and mortality, and the identification of precancerous lesions is regarded as a powerful tool for achieving this goal [1]. For example, potentially malignant disorders of the oral cavity (OPMDs) are associated with an increased risk of the occurrence of cancers of the oral cavity, and the detection of these precursor lesions has long been considered the gateway to early cancer diagnosis [2]. Disappointingly, global death rates for oral cancers have remained stagnant in the last 30 years [3], which stands in stark contrast to the declining trend of overall cancer death rates [4]. Epidemiological data also highlight that there is broad variation in oral cancer survival in relation to socio-demographic and geographical variables, a fact that likely reflects differences in healthcare access and risk factor exposure patterns. In particular, there has been a sharp increase in oral cancer incidence and mortality in recent decades in the Asia–Pacific region, a region where the areca nut economy has been booming [5]. This calls for policy changes and for a more imaginative yet evidence-based approach to tackling the oral cancer burden worldwide, particularly in high-risk populations.

In this article, I critically review the impact of OPMDs in the early detection of oral cancer and in reducing oral cancer burden, and make the case that some OPMDs may in fact represent overdiagnosis.

## 2. Epidemiology of Oral Cancer

It is not uncommon to come across different figures for what we colloquially refer to as oral cancer, for example, an age-standardized incidence rate between 4.1 and 7.1 per 100,000 for 2019/2020 has been reported [3,6] (Table 1). There are several reasons for this apparent inconsistency in the estimates.

Briefly, cancer data are primarily accessed via national or regional registries. For example, the Surveillance, Epidemiology, and End Results (SEERs) Program supported by the National Cancer Institute [7] is a major source of incidence and mortality data in the United States. Together with data from the National Program of Cancer Registries (NPCRs) established by the Centers for Disease Control and Prevention (CDC) [8], it covers the US population almost entirely [9]. Eventually, local data can be aggregated to produce estimates of cancer incidence, mortality, and prevalence worldwide, and GLOBOCAN [10] and the Global Burden of Disease (GBD) study [11] are two major sources of such global cancer statistics. Unfortunately, a sizeable number of countries do not have (accessible) cancer registries and, in these instances, data are derived from low-quality sources [12], with different strategies employed to overcome the lack of primary data, from approximating the rates to those of neighboring countries or registries in the same area to using “unusual data sources”. Further, it must be mentioned that while GLOBOCAN and GBD contribute significantly to our understanding of cancer epidemiology, these databases employ a variety of distinct and reasonable statistical models to generate their estimates [13,14], which inevitably leads to disparities in final cancer statistics. Even when using the same data source, differences may arise due to dissimilar statistical processing. For instance, the age-standardized mortality rate (ASMR) for lip and oral cavity (LOC) cancers reported in a recent analysis of the GBD study is 3.8 (per 100,000) [3], whereas it is 2.44 in Our World in Data [15], both utilizing the same IHME data source for cancer statistics.

Secondly, and somewhat surprisingly, while the anatomical district associated with oral cancer may appear straightforward, it is actually notably heterogeneous, and classifications can be confusing. For instance, the GBD data on lip, oral, and pharyngeal cancer [3] included ICD-10 codes C00 to C08 for lip and oral cavity cancers (including major salivary glands, but not oropharynx) and codes C09 to C10 and C12 to C13 for other pharyngeal cancers (OPC, including both oropharynx and hypopharynx, but not nasopharynx). Hence, it becomes challenging to infer the incidence and mortality for oral (and oropharyngeal) cancer unless specific coding data are made available. In contrast, GLOBOCAN and related publications [13] identify lip and oral cavity with ICD-10 codes C00 to C06 (thus excluding neoplasms of major salivary glands) and provide separate data for oropharynx (C09 and C10), making it easier to assess publicly available data related to oral and oropharyngeal cancers (Table 1). This, coupled with the differences in population coverage—and hence the representativeness and quality of the samples on which the estimates are based—explains the significant discrepancy observed between GLOBOCAN and GBD data [16,17]. For instance, in the United States, estimates of age-standardized incident cases of “lip and oral cavity” cancer vary between 6, 4.2, and 5.82 per 100,000, depending on the reporting source, either SEER, IARC (GLOBOCAN 2020), or IHME (GBD 2019), respectively. With regard to mortality, according to a recent analysis of the data from the GBD study 2019, the age-standardized LOC cancer death rate has remained unchanged at 3.8 per 100,000 worldwide over the past 30 years [3]. These data are particularly disappointing if one considers the emphasis placed by the dental profession on the early recognition of oral cancer and OPMDs.

Crucially, it has become clear that the epidemiology of oral cancer is following divergent paths. For example, in high-income countries where LOC cancer mortality has indeed reduced substantially (−20.4% ASDR), there has been a concomitant decrease in incidence (−14.2% ASIR), suggesting a genuine reduction in oral cancer cases, perhaps due to the increased awareness of the risk factors. In countries with a low or middle socio-demographic index (SDI), the increase in incidence has been paralleled by a comparable increase in mortality. In particular, in South and Southeast Asia, tobacco chewing contributed substantially to risk-attributable LOC cancer mortality according to the GBD study. Unfortunately, these data do not allow for an estimation of cancer deaths attributable to areca nut or betel quid chewing, as areca nut was not among the risk factors examined [18]. This omission represents a significant limitation of the GBD study, considering that areca nut is a well-known carcinogen and an independent risk factor for oral and oropharyngeal cancers, irrespective of tobacco use [19,20]. Strikingly, unlike tobacco chewers, those who chew BQ without tobacco experience a significant reversal in risk, making them ideal candidates for BQ cessation policies [21].

It is important, therefore, for research and policy to incorporate region-specific variations when addressing the burden of oral cancer [18]. These variations encompass not only differences in exposure to risk factors, such as betel quid use in South and Southeast Asia, but also disparities in the early diagnosis of the disease (secondary prevention), such as in the prevalence of precursor lesions and access to healthcare [22]. Understanding the impact of diagnosing OPMDs on the early detection of, and/or the reduction in mortality from, oral cancer is challenging due to the heterogeneity of data regarding the prevalence of OSCCs arising from OPMDs and the rate of the malignant transformation of OPMDs.

## 3. How Many Oral Cancers Are Preceded by an OPMD?

We became accustomed to the belief that a sizeable number of oral cavity cancers are preceded by OPMDs, but in fact this assumption is largely inaccurate. It is frequent to come across statements in the high-caliber peer reviewed literature that “*the majority* (of oral cancers) have a premalignant phase” [23], or that “*almost all* malignancies are developed from a clinically visible precursor stage called an OPMD” [24]. Contrary to this belief, several population-based studies have indicated that only a minority of OSCCs are associated with a diagnosis of precursor lesion, albeit with some geographical differences. For example, studies from the UK found that only 4.6% [25] and 6% [26] of oral cancers had arisen from previously recognized, site-specific precursor lesions that had been biopsied over a 12- and 20-year period, respectively. A higher prevalence of over 20% was recently found in European countries, such as Sweden [27] and Spain [28], although it is uncertain whether these OPMDs were biopsied and histopathologically proven. The figures derived from real-world evidence in Taiwan—a territory with one of the highest rates of OSCCs globally—are similar, with the prevalence of OSCCs preceded by OPMDs ranging between 9.9% and 15.7% (depending on how the data are interpreted) [29]. In Latin America, a recent retrospective observational study conducted in 17 centers reported that 18.6% of OSCCs had precursor lesions, the majority of which being leukoplakia [30]. Leukoplakia—reportedly the most common OPMD globally together with oral submucous fibrosis—was recognized as the precursor lesion in 7% of OSCCs in North America [31]. From these recent studies, it would appear that the vast majority of OSCCs arise de novo or at least without an identifiable precursor lesion.

A large-scale study in Ontario attempted to shed light on this important point by reviewing a series of 10,987 OSCCs to determine the proportion of OSCCs that were associated with preceding OPMDs and compare the outcome of the OSCC with or without a precursor [32]. Of all the oral and oropharyngeal cancer cases, only 3.44% had a preceding OPMD, and this frequency increased to 6.26% when the oropharynx was excluded.

While the Ontario study confirmed that the OSCCs that develop from OPMDs are a small minority, it could still be important to promptly detect these precursor lesions if OPMD diagnosis resulted in better prognosis for OSCCs. In this same study, of the OSCC patients with precursor lesions, 22.33% died of disease as opposed to 29.02% of those without a precursor lesion. One would be tempted to conclude that the early detection of OSCCs owing to an OPMD diagnosis can improve the outcome of oral cancer, but there are alternative explanations. First, the fact that patients with an OSCC associated with a precursor have significantly lower odds of dying from disease could be due to a different natural history of OSCC arising from OPMDs and, specifically, to less aggressive behavior. Secondly, individuals diagnosed with OPMDs are routinely monitored over time; hence, the survival of patients diagnosed with early-stage disease owing to an OPMD diagnosis is likely overestimated due to lead time bias.

Even if the survival difference found in the Ontario study was uniquely attributable to early cancer detection in OPMD patients (rather than to a different natural history of OSCC arising from OPMD), those figures translate into almost 21 lives potentially saved out of 10,394 OSCC patients with a known outcome, with an OPMD-population-attributable fraction for OSCC deaths of 0.7%. Even assuming that the death rates reported above did not overestimate the survival of individuals diagnosed with early-stage disease owing to an OPMD (despite lead time bias being a common weakness of survival estimates in studies concerned with early diagnosis [33]), from a healthcare perspective, the estimated reduction in oral cancer deaths attributable to OPMD monitoring (0.7%) is not overwhelming, particularly for a cancer for which the lifetime risk of death is overall around 1%.

In summary, only a small proportion of oral cancers are preceded by an observable OPMD, and the impact of OPMD-prompted early diagnosis on survival is questionable. Hence, according to the available evidence, the slight overall improvement in OSCC survival rates determined by OPMD diagnosis, if at all present, likely has a limited public health impact. As a prominent oral medicine and oncology specialist puts it, “from an individual’s point of view, early diagnosis is a must; however, from the public health view, it is a measure of probability. These perspectives are often at odds” [34]. This is likely to be the case for OPMDs.

## 4. How Many OPMDs Will Become Cancers?

The answer to this second point—how many potentially malignant diseases will in fact transform into cancer—largely depends on the length of the follow-up of individual studies. As a group, OPMDs have been reported to have a cumulative malignant transformation rate of 7.9% (99% CI 4.9–11.5%) based on a meta-analysis of 37,393 patients [35], a figure in agreement with the results of a most recent retrospective study of 5036 patients (cumulative malignant transformation of 6.4% with mean time for cancer development of 51.2 months) [36]. However, OPMDs include a number of clinically heterogeneous conditions, and the malignant transformation rates of individual diseases can vary widely (Table 2). For the OPMDs most commonly studied, the average cumulative transformation rates reported in recent systematic reviews range from 43.87% to 65.8% for PVL [37,38,39], 12.7% (19.9% in the meta-analysis) for erythroplakia [40], 7.20% to 9.8% for leukoplakia [41,42,43], 4.2% to 6% for oral submucous fibrosis [44,45], and 0.44% to 3.80% for oral lichen planus and lichenoid-type disorders [46,47,48], although the latter group is significantly influenced by the diagnostic criteria used [49], particularly the inclusion of dysplasia [50]. These estimates seem to be relatively homogeneous worldwide, including Asian countries, although there are differences reflecting regional risk factors. In a prospective cohort study in Taiwan, the yearly rate of transformation for OPMDs was 0.84% (with rates as high as 3.3% for verrucous leukoplakia) [51]. In a national-wide oral screening of 3,362,232 people in Taiwan, the malignant transformation rate was 24.55, 12.76, 9.75, and 4.23 per 1000 person years in the verrucous hyperplasia, oral submucous fibrosis, erythroplakia, and leukoplakia cohorts [52].

To make different studies comparable, it would be important to report the yearly incidence of transformation whenever possible. In this regard, a meta-analysis calculated that the annual malignant transformation rates (AMTRs, based on the average follow-up as reported in the various subgroups) were lowest for lichen planus (0.28%) and oral lichenoid lesions (0.57%) and highest for erythroplakia (2.7%) and PVL (9.3%). Mild dysplasia had an annual malignant transformation of 1.7%, while that of severe dysplasia was 3.57% [35].

From these data, oral conditions with a risk of malignant transformation equal to or greater than ~1% include proliferative leukoplakia (often referred to as PVL, although this term may be misleading) erythroplakia, and leukoplakia, whereas for all other OPMDs, the risk is in fact relatively low in the absence of epithelial dysplasia. The only OPMD with considerable intra-disease variability, and hence uncertain risk, is oral leukoplakia. A systematic review of observational studies that focused specifically on oral leukoplakia reported a mean malignant transformation rate of 3.5% among 11,423 patients (range between 0.13% and 34.0%) [42], where size (lesion exceeding 200 mm^2^) and type (non-homogeneous) were significant determinants contributing to the malignant potential of leukoplakia. Of the 24 studies included in the systematic review [42], the annual transformation rate was only offered for three articles, one of which stands out for its relatively high AMTR of 6.9%. At a closer look, however, the study in question by Saito et al. [53] showed that the lesions that exhibited a high (25%) transformation rate over ~4 years were widespread multiple oral leukoplakias, which presumably could have been classified as proliferative leukoplakia. In fact, in this same study, localized leukoplakia had an annual transformation rate as low as 0.6%. In an updated systematic review and meta-analysis of studies published between 2015 and 2020, the crude pooled transformation rate was 6.77% (which further dropped to 5.63% in higher-quality studies) [54]. Again, no yearly rate of transformation was calculated, and given a follow-up range of between 1.8 and 21.6 years in these studies, it is not implausible to conclude that the AMTR was below 1%.

**Table 2:** List of oral potentially malignant disorders with a quantifiable rate of malignant transformation. *CMTR*, cumulative malignant transformation rate; *AMTR*, annual malignant transformation rate. The table provides a snapshot of representative single percentages from large meta-analyses; see text for more details. Where AMTR was not provided, it was approximated using the mean follow-up reported in the studies included, where available. Alternatively, a different source was used and referenced.

Disease	CMTR	AMTR	n. of Patients	Ref.
Proliferative leukoplakia	45.8%	6.36%	699	[38]
Erythroplakia	12.7%	1.91%	441	[40]
Leukoplakia	9.7%	1.35%	23,489	[43]
Oral submucous fibrosis	4.2%	0.73%	6337	[44]
Oral lichen planus	0.94%	0.28% [35]	22,578	[49]
Oral lichenoid lesions ^#^	1.95%	0.35% [55]	717	[49]
Actinic keratosis/cheilitis ^##^	14%	1.69% [56]	728	[57]
Chronic hyperplastic candidiasis ^##^	12.1%	1.51%	274	[58]

^#^ denotes being added to and ^##^ removed from the 5th Edition of the World Health Organization Classification of Head and Neck Tumors 2022 [59].

In summary, the evidence shows that a minority of oral cancers, presumably between 5% and 20%, are preceded by OPMDs, and that most OPMDs have an annual transformation rate <1%. Thus, while looking harder for oral cancer in OPMD patients does not seem to be returning the expected yield, it still turns millions of individuals into may-be cancer patients requiring life-long monitoring. This is fertile ground for overdiagnosis and overtreatment to flourish.

## 5. Overdiagnosis of OPMDs

An OPMD is defined as “any oral mucosal abnormality that is associated with a statistically increased risk of developing oral cancer” [2]. However, the evidence behind the inclusion in or exclusion from the OPMD list is not entirely clear, nor is the methodology and process leading to this consensus (e.g., what guidelines for reporting consensus-based methods were used; how consensus was reached). Except for the “big six” (Table 2), data describing the malignant transformation of other OPMDs are scant; hence, it is technically impossible to demonstrate a “statistically increased risk” of transformation. Remarkably, the list of OPMDs included in the most recent WHO Classification of Head and Neck Tumors [59] differs from the one available from consensus documents [2], adding further confusion.

Overdiagnosis occurs when individuals are diagnosed with conditions that will never cause symptoms or death [33] and typically presents when incident cases increase significantly over a period of time while late-stage disease and/or mortality remain largely unchanged. At a global level, the age-standardized incidence rate (ASIR) of LOC increased only slightly (5.4%, from 6.7/100,000 to 7.1/100,000) from 1990 to 2019, whereas the death rate remained unchanged, suggesting that the overdiagnosis of LOC cancers may be occurring, but not on a large scale. But that does not mean that there is no overdiagnosis; there may be the overdiagnosis of precancerous abnormalities, namely OPMDs, as these disorders are extremely common.

A meta-analysis showed that the global prevalence of OPMDs is 4.47% (excluding oral lichen planus and related disorders), with rates as high as 10.54% in Asia [60], and oral lichen planus occurs in about 1% of the population [61]. These findings align with our pilot population study surveying five Indonesian provinces, where over 14% of participants had an OPMD [62], with leukoplakia being the most common. In a more recent meta-analysis, the global prevalence of leukoplakia was found to be 1.39%, with a range from 0.12 to 33.33% [63].

These numbers are astonishing: nearly half a billion people worldwide have an oral disease that is identified as potentially progressing to cancer. One might wonder whether there is something wrong with these figures; if OPMDs had an annual transformation rate as low as 0.25% (which is lower than the annual transformation rate for any OPMD, as shown in Table 2), we would expect to see over 1 million new cases of OPMD-preceded oral cancers each year. However, the actual number of oral cancer cases preceded by precursor lesions is less than a tenth of that estimate. It is likely, therefore, that either the prevalence of OPMDs or the risk of malignant transformation, or perhaps both, are overestimated. Alternatively, there is a vast reservoir of undiagnosed oral cancers worldwide.

Labeling such a large segment of the population as abnormal and in need of treatment is a serious concern, particularly when only a small fraction may actually benefit. The majority of patients with ‘precursor’ lesions are likely not on a path to developing cancer, making a diagnosis of an OPMD potentially more harmful than helpful. The ‘minor’ harms associated with the anxiety of a precancer diagnosis, rigorous follow-ups, and invasive treatments can become significant when applied to a large population. For instance, recent studies indicate that the diagnosis and subsequent management of an OPMD can lead to considerable out-of-pocket expenses, resulting in catastrophic health expenditure for households [64]. Moreover, the psychological impact on these patients, in terms of depression, anxiety, and stress, is notable [65], and strategies to improve communication with patients have been proposed accordingly [66]. Therefore, the decision to inform a patient that their OPMD could potentially progress to cancer one day must not be taken lightly and should be made with consideration of the broader implications.

Here, I question the need to consider some of these conditions as potentially malignant in the first place. As noted in the latest IARC handbook, numerous follow-up studies have shown that over time, OPMDs may remain stable, change in size, or even completely resolve [67]. As we have seen above, except for a few clinically defined cases, the proportion of OPMDs that progress to life-threatening oral cancers is actually quite small when viewed in a broader context. Therefore, while a diagnosis of a disease regarded as potentially malignant may benefit a limited number of individuals, the majority of patients may unnecessarily bear the burden of a ‘precancer label’ and potentially undergo unnecessary treatments. It can be argued that the only patients with oral mucosal diseases at a higher risk of malignant transformation are those with proliferative leukoplakia, erythroplakia, and possibly non-homogeneous leukoplakia.

## 6. Conclusions

The approach to oral cancer prevention and management has long emphasized the role of identifying and treating OPMDs. However, this strategy appears to be insufficient in addressing the persistent and unchanged death rates from lip and oral cavity cancer over the past three decades. The current understanding that only a small fraction of oral cancers are preceded by OPMDs and the low annual transformation rate of most OPMDs into cancer suggest the need for a revised approach.

Given the heterogeneity and varying risk profiles of precursor lesions, it is crucial to focus intervention efforts on those with a significant risk of malignant transformation, rather than broadening the scope to all OPMDs. Hence, the strategy of considering all OPMDs as potential harbingers of malignancy needs re-evaluation. Instead, a more selective approach that targets specific high-risk conditions, coupled with effective global public health strategies focusing on education and prevention, especially in high-risk and high-incidence regions, could be more impactful.

Ultimately, reducing the global burden of oral cancer requires a multifaceted approach that includes redefining the criteria for potentially malignant disorders, focusing on high-risk conditions and early manifestations of oral cancers (whether or not related to precursor lesions), improving education for dental professionals, and implementing effective public health policies. Such a strategy, rooted in evidence-based practice and tailored to regional variations in risk factors and incidence, may provide a more effective path toward decreasing the incidence and mortality of oral cancer.

## Figures and Tables

**Table 1:** List of International Classification of Diseases (ICD) codes mapped to the Global Burden of Disease 2019 and Global Cancer Observatory (GLOBOCAN) 2020 for lip and oral cavity cancer and other pharynx cancer. *ASIR*, age-standardized incidence rate; *ASMR*, age-standardized mortality rate (per 100,000 people).

	Global Burden of Disease 2019		GLOBOCAN 2020	
ICD-10 Codes	ASIR (95% UI)	ASMR (95% UI)	ASIR	ASMR
Lip and oral cavity (C00–06)	7.1 (6.5–7.7)	3.8 (3.5–4.2)	4.1	1.9
Salivary glands (C07, C08)			0.57	0.23
Oropharynx (C09, C10)	3.2 (2.9–3.4)	2.2 (2.0–2.4)	1.1	0.51
Hypopharynx (C12, C13)			0.91	0.41
